# Effects of Standardized Green Tea Extract and Its Main Component, EGCG, on Mitochondrial Function and Contractile Performance of Healthy Rat Cardiomyocytes

**DOI:** 10.3390/nu12102949

**Published:** 2020-09-25

**Authors:** Rocchina Vilella, Gianluca Sgarbi, Valeria Naponelli, Monia Savi, Leonardo Bocchi, Francesca Liuzzi, Riccardo Righetti, Federico Quaini, Caterina Frati, Saverio Bettuzzi, Giancarlo Solaini, Donatella Stilli, Federica Rizzi, Alessandra Baracca

**Affiliations:** 1 Department of Chemistry, Life Sciences and Environmental Sustainability (SCVSA), University of Parma, 43124 Parma, Italy; rocchina.vilella@studenti.unipr.it (R.V.); monia.savi@unipr.it (M.S.); leonardo.bocchi@unipr.it (L.B.); donatella.stilli@unipr.it (D.S.); 2 Department of Biomedical and Neuromotor Sciences (DIBINEM), Laboratory of Biochemistry and Mitochondrial Pathophysiology, University of Bologna, 40126 Bologna, Italy; gianluca.sgarbi@unibo.it (G.S.); francesca.liuzzi4@unibo.it (F.L.); giancarlo.solaini@unibo.it (G.S.); 3 Department of Medicine and Surgery (DIMEC), University of Parma, 43125 Parma, Italy; valeria.naponelli@unipr.it (V.N.); federico.quaini@unipr.it (F.Q.); caterina.frati@unipr.it (C.F.); saverio.bettuzzi@unipr.it (S.B.); 4 National Institute of Biostructure and Biosystems (INBB), 00136 Rome, Italy; 5 Centre for Molecular and Translational Oncology (COMT), University of Parma, 43124 Parma, Italy; 6 CNR Institute of Molecular Genetics “Luigi Luca Cavalli-Sforza” Unit of Bologna, 40136 Bologna, Italy; riccardo.righetti@studio.unibo.it

**Keywords:** cardiomyocyte mechanics, green tea extracts, mitochondria, oxidative phosphorylation, phospholamban, SERCA2

## Abstract

We recently showed that the long-term in vivo administration of green tea catechin extract (GTE) resulted in hyperdynamic cardiomyocyte contractility. The present study investigates the mechanisms underlying GTE action in comparison to its major component, epigallocatechin-3-gallate (EGCG), given at the equivalent amount that would be in the entirety of GTE. Twenty-six male Wistar rats were given 40 mL/day of a tap water solution with either standardized GTE or pure EGCG for 4 weeks. Cardiomyocytes were then isolated for the study. Cellular bioenergetics was found to be significantly improved in both GTE- and EGCG-fed rats compared to that in controls as shown by measuring the maximal mitochondrial respiration rate and the cellular ATP level. Notably, the improvement of mitochondrial function was associated with increased levels of oxidative phosphorylation complexes, whereas the cellular mitochondrial mass was unchanged. However, only the GTE supplement improved cardiomyocyte mechanics and intracellular calcium dynamics, by lowering the expression of total phospholamban (PLB), which led to an increase of both the phosphorylated-PLB/PLB and the sarco-endoplasmic reticulum calcium ATPase/PLB ratios. Our findings suggest that GTE might be a valuable adjuvant tool for counteracting the occurrence and/or the progression of cardiomyopathies in which mitochondrial dysfunction and alteration of intracellular calcium dynamics constitute early pathogenic factors.

## 1. Introduction

Tea is the second most commonly consumed beverage in the world after water [1]. A water infusion of the dried leaves of *Camellia sinensis* is widely known as green or black tea. Green tea is unique because fresh harvested leaves must be quickly heat dried to prevent the enzymatic oxidation and polymerization of the catechins, a family of polyphenols that constitute the major component of green tea. The most abundant catechins in green tea are (−)-epigallocatechin-3-gallate (EGCG), (−)-epigallocatechin (EGC), (−)-epicatechin-3-gallate (ECG), and (−)-epicatechin (EC). In a typical green tea extract (GTE), EGCG is believed to be the major factor responsible for the biological effects [2,3,4,5]. Among the many biological effects attributed to green tea, catechins were found to be associated with potential anticancer activity [6,7,8], to interact with gut microbiota [9,10], and to protect against nonalcoholic steatohepatitis [11,12]. More interestingly, for the purpose of the present work, green tea catechins improved cardiovascular and metabolic health [13,14,15,16], one aspect of which was the provision of protection against diet-induced obesity [17]. Mechanisms involving reactive oxygen species (ROS) scavenging and reduction of tissue inflammation [18,19], mitochondrial function improvement, and transcriptional control of genes involved in the adaptive response to oxidative stress [20] have been reported.

We recently demonstrated that the long-term in vivo oral administration to healthy rats of a standardized GTE (Theaphenon-E^TM^) increased contractile efficiency and intracellular calcium dynamics in cardiomyocytes by enhancing the sarcoplasmic reticulum calcium-ATPase (SERCA2) activity and the cell energy availability, thus suggesting that mitochondria are a possible target of GTE [21].

Mitochondria provide virtually all of the heart’s ATP through oxidative phosphorylation (OXPHOS), a process occurring at the inner mitochondrial membrane where the four complexes of the respiratory chain (Complex I, II, III, and IV) and the ATP synthase (F_1_F_0_-ATPase) are located. Under physiological conditions (i.e., normoxia and moderate hypoxia), the respiratory chain complexes oxidize NADH and FADH_2_, reducing molecular oxygen; this exergonic process induces the formation of a transmembrane electrochemical potential (Δμ*_H_*+) that is used by the ATP synthase to catalyze the condensation of inorganic phosphate with ADP to produce ATP [22]. Changes of several intracellular pathways involved in ATP supply, ROS production, mitochondrial biogenesis, mitophagy, and the release of pro-apoptotic factors from mitochondria may contribute to both the etiology and/or the progression of many diseases [23,24,25,26,27]. Among these, cardiomyopathies have also been associated with early mitochondrial dysfunction [28,29]. Therefore, we sought to find out whether mitochondria could be the target of GTE and EGCG action.

Based on our previous findings [21], the aim of the present study was twofold. First, we investigated in-depth the mitochondrial mechanisms involved in the enhanced energy availability observed in cardiomyocytes from healthy GTE-supplemented rats. Secondly, we studied whether the effects of GTE on mitochondrial function and contractile performance can be entirely attributed to epigallocatechin-3-gallate (EGCG), its major bioactive component. To reduce possible biases and interexperimental variability, we used the standardized mixture called Theaphenon-E^TM^ as the source of GTE.

## 2. Materials and Methods

### 2.1. Ethics Approval

The investigation was approved by the Veterinary Animal Care and Use Committee of the University of Parma, Italy (Prot. No. 614/2016-PR) and conforms to the National Ethical Guidelines of the Italian Ministry of Health and the Guide for the Care and Use of Laboratory Animals (National Institute of Health, Bethesda, MD, USA, revised 1996). All experiments were carried out in accordance with the approved guidelines. Every effort was made to minimize animal suffering.

### 2.2. Animals and Experimental Protocol

The study population consisted of 26 male Wistar rats (*Rattus norvegicus*) aged 16–18 weeks, weighing 425.5 ± 6.3 g, individually housed in a temperature-controlled room at 22–24 °C in which the light was kept on between 7 a.m. and 7 p.m. The bedding of the cages consisted of wood shavings, and food was freely available (Mucedola s.r.l., Milan, Italy). The animals were randomly divided into three experimental groups: (i) the GTE group: 9 rats receiving 40 mL/day of tap water containing a standardized green tea extract, Theaphenon-E (from Tea Solutions, Hara Office Inc., Japan) at a concentration of 0.3% *w/v*; (ii) the EGCG group: 9 rats receiving 40 mL/day of a tap water solution of EGCG (epigallocatechin-3-gallate Sigma-Aldrich, Milan, Italy) at the same dosage as in GTE (i.e., 0.21% *w/v*; 300 mg of Theaphenon E dry powder contains 210 mg of EGCG); (iii) the CTRL group: 8 rats receiving 40 mL/day of tap water. Theaphenon-E is a dried powder that has been stored in the dark and under moisture-controlled conditions. A solution that was freshly prepared in tap water was orally administered every day to the experimental animals in dark bottles to prevent oxidation [30]. We gave 40 mL/day to each rat because this volume corresponds to the lowest value of the range of daily water consumption in male rats, which were matched for age and body weight, as previously reported [21]. The concentration of Theaphenon-E in drinking water that was chosen for the study is the maximum amount of catechins in a water solution whose taste is well tolerated by rats and that does not cause side effects. This protocol has been used extensively in the past by our research team and other investigators [6,21,31]. Theaphenon-E is composed of the following: epigallocatechin gallate (68.58%), epigallocatechin (10.56%), epicatechin gallate (5.95%), epicatechin (4.31%), gallocatechin gallate (1.23%), DL-catechin (0.56%), caffeine (0.44%), and traces of theobromine and gallic acid [32,33]. Specifically, catechins are purified from green tea leaves by extraction with water first and then with ethyl acetate, followed by column chromatography, using water/alcohol to elute the catechins. The resulting catechin powder has a total catechin content of about 90% [33]. The rats’ body weight was measured every week until sacrifice. All animals showed a small weight gain over the duration of the experiment (approximately 3%). No statistically significant differences were found among the three experimental groups at all time increments (Table 1).

At day 28, animals were anesthetized with ketamine chloride (Imalgene, Merial, Milan, Italy; 40 mg/kg i.p.) plus medetomidine hydrochloride (Domitor, Pfizer Italia S.r.l., Latina, Italy; 0.15 mg/kg i.p.), and then used for experimental measurements.

The hearts of 15 animals (5 GTE, 5 EGCG, and 5 CTRL) were excised, and cardiac cells were enzymatically isolated, as previously reported [21]. Then, isolated cardiomyocytes were swiftly implemented for determining mechanical properties and calcium transients with no further treatment. A fraction of the isolated cardiomyocytes was washed three times with low-calcium (0.1 mM) saline solution and recovered by centrifugation at 500 rpm for 5 min. After removing the supernatant, the cell pellet was stored at −80 °C for the biochemical assays. Mitochondrial respiration measurements were performed in freshly isolated cardiomyocytes. The hearts of 8 rats (3 GTE, 3 EGCG, and 2 CTRL) were excised after perfusion with Tyrode’s solution; the left ventricles (LVs) were snap-frozen in liquid nitrogen and stored at −80 °C for subsequent molecular analyses. The hearts of the remaining animals were arrested in diastole by perfusion with CdCl_2_ (100 mM) and used for transmission electron microscopy analysis (TEM).

### 2.3. Measurement of Cardiomyocyte Mechanics and Ca^2+^ Transients

Isolated cardiomyocytes were suspended in low-calcium (0.1 mM) saline solution for 20 min, gradually brought to 1 mM Ca^2+^ in about 80 min, and then used for measuring calcium transients and sarcomere shortening. Mechanical properties and calcium transients were recorded by using the IonOptix fluorescence and contractility systems (IonOptix, Milton, MA, USA), as previously described [21]. Left ventricle (LV) myocytes were placed in a chamber mounted on the stage of an inverted microscope (Nikon-Eclipse TE2000-U, Nikon Instruments, Florence, Italy) and superfused (1 mL/min at 37 °C) with a Tyrode solution containing (in mM, all chemicals from Sigma-Aldrich): 140 NaCl, 5.4 KCl, 1 MgCl_2_, 5 HEPES, 5.5 glucose, and 1 CaCl_2_ (pH 7.4, adjusted with NaOH). Only rod-shaped myocytes with clear edges and average sarcomere length ≥ 1.7 µm were selected for the analysis. None of the selected myocytes showed spontaneous contractions. The cells were field stimulated at a frequency of 0.5 Hz by constant current pulses (2 ms in duration, and twice the diastolic threshold in intensity; MyoPacer Field Stimulator, IonOptix). A total of 66 CTRL cells, 72 GTE cells, and 78 EGCG cells were analyzed. Ca^2+^ transients were detected by epifluorescence after loading the myocytes with Fluo-3 AM (5 µM; Thermo Fisher Scientific, Waltham, MA, USA), previously mixed with Pluronic^TM^ F-127 (10% final concentration; Thermo Fisher Scientific, Waltham, MA, USA), for 20 min. Excitation length was 480 nm, with emission collected at 535 nm. The steady-state contraction of myocytes was achieved before data recording by means of a 10 s conditioning stimulation. The sampling rate was set at 1 kHz. The following parameters were computed: mean diastolic sarcomere length, fraction of shortening (FS), maximal rates of shortening (−dl/dt_max_) and relengthening (+dl/dt_max_), and time to 10%, 50%, and 90% of relengthening (RL10%, RL50%, and RL90%). Fluo-3 signals were expressed as normalized fluorescence (f/f0: fold increase). The time course of the fluorescence signal decay was described by a single exponential equation, and the time constant (tau) was used as a measure of the rate of intracellular calcium clearing [34].

### 2.4. ATP Content Analysis in Isolated Cardiomyocytes

The ATP intracellular content was measured by the Luminescence ATP Detection Assay System (ATPlite; PerkinElmer, Waltham, MA, USA) according to the manufacturer’s protocol. Briefly, a frozen pellet of isolated LV cardiomyocytes was resuspended in 1 mL of Phosphate Buffered Saline (PBS); 20 μL of this suspension was further diluted to a final volume of 400 μL with PBS. Aliquots of 100 μL of the diluted cell suspension were pipetted in triplicate in a 96-well white plate, lysed with 50 μL of mammalian cell lysis solution for 5 min in an orbital shaker, and then added to 50 μL/well of substrate solution. The microplate was shaken again and dark-incubated for 10 min. Once the incubation time expired, the luminescence intensity was measured by the EnSpire^®^ multimode plate reader (PerkinElmer). The row luminescence data, given in relative light units (RLUs), were normalized to the total protein content of each sample, measured by the DC Protein assay kit (Bio-Rad, Hercules, CA, USA).

### 2.5. Respiration Rate Measurements

The respiratory rate of control and supplemented rat cardiomyocytes was assayed polarographically using a Clark-type oxygen electrode in a buffer containing 250 mM sucrose, 20 mM Tris/Cl, 4 mM MgSO_4_, 0.5 mM EDTA, and 10 mM KH_2_PO_4_, pH 7.4. Under state 3 conditions, the NADH-dependent oxygen consumption was measured at 30 °C (saturating oxygen concentration is 204.1 µM) in permeabilized cells (60 µg/mL digitonin; 1.5 × 10^5^ cells/mL) by adding 10 mM glutamate, 10 mM malate (plus 1.8 mM malonate), and 0.5 mM ADP as substrates [23]. The state 4 respiration rate was measured following the addition of 1 µM oligomycin [35]. We calculated the initial rate of respiration under both state 3 and state 4 conditions by evaluating the oxygen concentration decline during the first two minutes of reaction of each state. The respiratory control ratio (RCR) was defined as the state 3/state 4 respiratory rates’ ratio. Cells were counted by using the MUSE cell analyzer (Millipore, Billerica, MA, USA), and the respiration rates were expressed as nmol/min/10^6^ cells.

### 2.6. Citrate Synthase Activity Assay

The citrate synthase activity of rat cardiomyocytes was detected by incubating 1.5 × 10^3^ cells in 1 mL of 0.125 M Tris-HCl, 0.2% triton, 0.1 mM acetyl-coenzyme A, 0.1 mM 5,5′-dithio-bis (2-nitrobenzoic acid (DTNB), and 0.5 mM oxaloacetate. The activity was assessed by monitoring the release of 2-nitro-5-thiobenzoate (ε = 13.6 mM^−1^cm^−1^) at 412 nm and was expressed as µmol/min/10^6^ cells [36].

### 2.7. DNA Extraction and Relative Quantification of the mtDNA Content

Total DNA was extracted from GTE, EGCG, or CTRL rat left ventricles (LVs) by using the commercial Kit NucleoSpin Tissue (Macherey-Nagel, Duren, Germany) according to manufacturer’s instructions and quantified by a Biospectrophotometer (Eppendorf AG, Germany). Then, 0.1 ng of DNA, quantified by the QuantiFluor ONE System using the Quantus Fluorometer (Promega Corporation, Madison, USA), was used for qPCR amplification by the SSO-Advanced Universal SYBR Green Supermix (Bio-Rad). Mitochondrial DNA (mito gene) corresponding to sequences of the NADH-ubiquinone oxidoreductase subunit 1 (*ND1*) and the NADH-ubiquinone oxidoreductase subunit 3 (*ND3*) was amplified using the primer set shown in Table 2. Primers were carefully chosen in order to avoid amplification of nuclear mitochondrial insertion sequences (pseudogenes) that might negatively affect accurate mtDNA quantification. The *GAPDH* gene was used as a nuclear DNA marker (nucl gene). Thermal cycler conditions consisted of an initial denaturation at 95 °C for 30 s, followed by 40 cycles of denaturation at 95 °C for 15 s, and annealing and extension at 60 °C for 20 s. To calculate the relative mtDNA content, the following equations were used:

ΔCt = Ct _nucl gene_ − Ct _mito gene_; Relative mtDNA content = 2 × 2^ΔCt^ (1)

### 2.8. RNA Extraction, cDNA Preparation, and Relative Expression of Genes Involved in Mitochondrial Biogenesis

GTE, EGCG, or CTRL rat LVs were ground in liquid nitrogen to a fine powder. For RNA extraction, 10 mg of tissue was lysed with 1 mL of TRIZOL reagent (Thermo Fisher Scientific, Waltham, MA, USA), purified with the PureLink RNA Mini kit (Thermo Fisher Scientific), and reverse transcribed using the RevertAid First Strand cDNA Synthesis kit (Thermo Fisher Scientific) according to the manufacturer’s instructions. Briefly, for each sample 250 ng of total purified RNA was combined with 1 µL of Oligo dT Primers (100 µM) and heated at 65 °C for 5 min. After a quick chilling on ice, the first strand synthesis reaction was carried out for 60 min at 42 °C and stopped at 70 °C for 5 min. Two microliters of the cDNA obtained was used for qPCR amplification by the SSO-Advanced Universal SYBR Green Supermix (Bio-Rad). Each cDNA sample was run in duplicate on the DNA Engine Opticon 4 (MJ Research, Waltham, MA, USA). Thermal cycler conditions consisted of an initial denaturation at 95 °C for 30 s, followed by 40 cycles of denaturation at 95 °C for 15 s, and annealing and extension at 60 °C for 20 s. The primers used for the amplification of peroxisome proliferator-activated receptor gamma coactivator 1-alpha (*PGC-1α*), nuclear respiratory factor 1 (*NRF1*), and mitochondrial transcription factor A (*TFAM*) are shown in Table 2. qPCR data analysis was performed by calculating the cycle threshold (Ct) for each gene, where Ct is the number of cycles required for the fluorescent signal to cross the threshold. Row Ct values of target genes were normalized to the Ct value of the reference gene glyceraldehyde phosphate dehydrogenase (*GAPDH*) according to the following equation:

normalized Ct (ΔCt) = Ct _target gene_ − Ct _GAPDH_. (2)

### 2.9. Protein Extraction, SDS–Polyacrylamide Gel Electrophoresis (SDS-PAGE), and Western Blot (WB) Analysis

GTE, EGCG, and CTRL rat LVs were finely ground in liquid nitrogen, then 30 mg of powder was homogenized in 500 µL of ice-cold Radioimmunoprecipitation Assay Buffer (RIPA) supplemented with adequate amounts of protease and phosphatase inhibitor cocktails (Sigma-Aldrich, Milan, Italy). In addition, ice-cold RIPA buffer plus inhibitors was used to lyse isolated cardiomyocytes. Thirty micrograms (3–6 µg when OXPHOS complexes were analyzed) of protein lysate was separated by SDS-PAGE, blotted onto PVDF or nitrocellulose membranes, and quantified by immunodetection analysis. After blocking in a solution of 2% bovine serum albumin, the membranes were incubated with primary antibodies: rabbit polyclonal anti-phospho-phospholamban (Ser16) (EMD Millipore Corporation, Temecula, CA, code 07-052), dilution 1:200; mouse monoclonal anti-phospholamban (2D12), (Abcam, Cambridge, UK, code ab2865), dilution 1:1000; rabbit polyclonal anti-SERCA2 ATPase, (Abcam, Cambridge, UK, code ab3625), dilution 1:1000 mouse monoclonal anti-actin (Santa-Cruz Biotechnology, Santa Cruz, CA, USA, code sc-81178) dilution 1:500 [21].

The antibodies used to label the OXPHOS complexes were the following: anti-NDUFA9 (NADH:ubiquinone oxidoreductase subunit A9), dilution 1:1000, for Complex I; anti-SDHA (succinate dehydrogenase complex flavoprotein subunit A), dilution 1:2500, for Complex II; anti-UQCRC2 (ubiquinol-cytochrome c reductase core protein 2), dilution 1:1000, for Complex III; anti-COX-I (cytochrome c oxidase subunit I), dilution 1:1000, for Complex IV; anti-ATP5A (ATP synthase F_1_ subunit alpha), dilution 1:2000, and anti-ATP5H (ATP synthase F_1_ subunit d), dilution 1:1000, for Complex V. All the antibodies were purchased from AbCam (Cambridge, UK; catalog numbers are ab14713, ab14715, ab14745, ab14705, ab14748, and ab110275, respectively) and used as previously described [40]. As a loading control, β-actin (Santa-Cruz Biotechnology, Santa Cruz, CA, USA, 1:500, sc-81178) or the translocase of the outer mitochondrial membrane 20 (TOMM20) (AbCam, Cambridge, UK; 1:1000, ab56783) was used. Detection of the immunoreactive bands was achieved using horseradish peroxidase-conjugated anti-mouse (dilution 1:5000) or anti-rabbit (dilution 1:200,000) secondary antibodies (Sigma-Aldrich, Milan, Italy, catalog numbers are A5906 and A0545, respectively) and the BM Chemiluminescence Blotting Substrate (POD) (Hoffmann-La Roche, Basel, Switzerland Catalog number 11 500 694 00).

### 2.10. Ultrastructural Analysis of LV Myocardium by TEM

Fresh LV samples were fixed in Karnovsky, washed several times with phosphate buffer (0.1 M, pH 7.2), and post-fixed for 90 min at Room Temperature (RT) in 1% osmium tetroxide (OsO_4_). Following dehydration achieved by increasing the concentration of alcohol, samples were washed with propylene oxide and embedded in epoxy resin. Sections of 0.5 μm thickness were stained with methylene blue and safranin in order to select the field of interest. Ultrathin sections (60–80 nm thickness) were collected on a 300-mesh copper grid and stained with uranyl acetate and lead citrate. Sections were qualitatively examined under a transmission electron microscope (Philips EM 208S; Fei Electron Optics BV, Eindhoven, Netherlands).

### 2.11. Statistical Analysis

The IBM SPSS statistical package (International Business Machines Corporation, Armonk, NY, USA, version 25) was used. Normal distribution of variables was checked by means of the Kolmogorov–Smirnov test. Data are expressed as mean values ± standard error of the mean (SEM). Comparisons among groups involved GLM ANOVA for repeated measurements (cell mechanics, Ca^2+^ transients, and intracellular ATP levels) followed by Sidak post hoc test, one-way ANOVA followed by the Bonferroni’s post hoc test (mitochondrial respiration rates), and non-parametric median test (mtDNA content, mitochondrial biogenesis, and protein expression). Two-tailed *p* values less than 0.05 were regarded as statistically significant.

## 3. Results

### 3.1. ATP Content and Mitochondrial Respiration in Cardiomyocytes of GTE- and EGCG-Supplemented Rats

**Figure 1:**
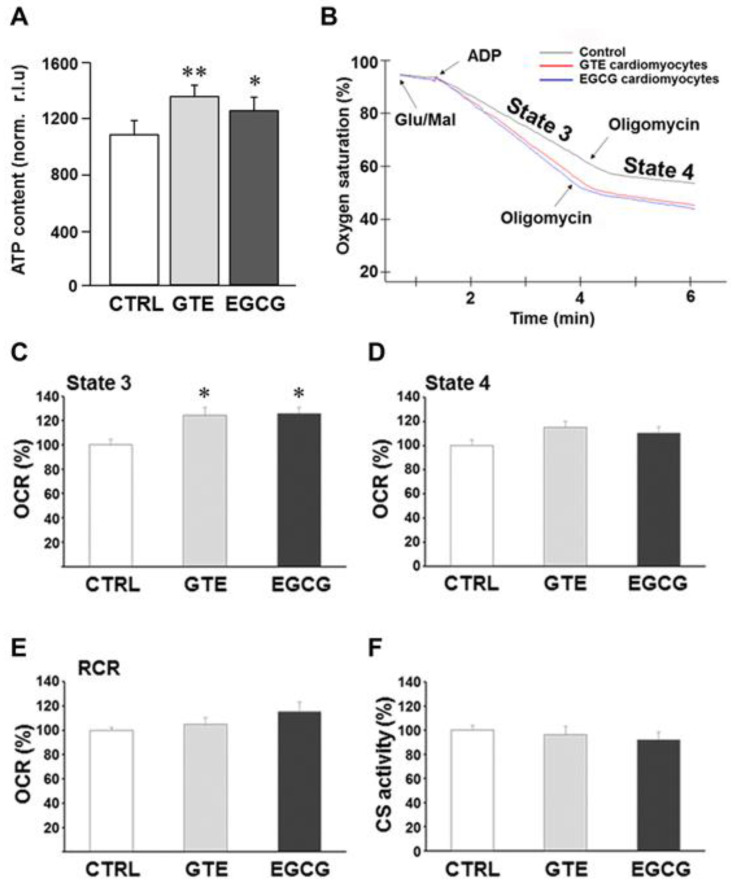
Effects of GTE and EGCG administration on rat cardiomyocyte ATP content and mitochondrial respiration. (**A**) ATP content of cardiomyocytes isolated from left ventricle (LV) myocardium of CTRL (*n* = 5), GTE- (*n* = 5) and EGCG-supplemented (*n* = 5) rats, expressed as relative light units (RLUs) normalized to the protein content. (**B**) Typical complex I-driven oxygen consumption traces of permeabilized cardiomyocytes under different respiratory conditions. Respiration rates under state 3 (**C**) and state 4 (**D**) conditions expressed as percent of controls. (**E**) Respiratory control ratios (RCRs) (state 3/state 4 respiration rate ratio) were reported as percent of controls. State 3 and state 4 respiration rates of controls were 189.19 ± 10.37 and 41.38 ± 3.02 nmol/min/10^6^ cells, respectively, and RCR was 4.59 ± 0.14. (**F**) The citrate synthase activity of GTE and EGCG rat cardiomyocytes was reported as percent of controls (7.68 ± 0.30 µmol/min/10^6^ cells). Histograms show the mean ± SEM. * *p* < 0.05 and ** *p* < 0.01 indicate the statistical significance of data compared to controls. CTRL = Control; GTE = green tea catechin extract; EGCG = epigallocatechin-3-gallate.

We first evaluated the effect of GTE and EGCG on rat-cardiomyocyte ATP levels (Figure 1A). A significant increase of the steady-state ATP content was measured in both GTE (+25%) and EGCG (+16%) rat cardiomyocytes as compared to that in controls (CTRL).

To examine whether, and how, mitochondrial function contributed toward the improvement of the energy availability in cardiomyocytes isolated from GTE- or EGCG-supplemented rats, we analyzed in-depth the oxygen consumption rate (i.e., respiratory chain activity). Complex I-dependent respiration measurements were carried out in permeabilized cells under both state 3 and state 4 respiration conditions; the assay was carried out in the presence of ADP and glutamate/malate as the energizing substrates (state 3 or maximal respiration rate). Oligomycin was added to the reaction mixture to measure the state 4 respiration rate (Figure 1B). A significant increase (+25%) of the maximal respiration rate was measured in both GTE and EGCG cardiomyocytes compared to that in controls (Figure 1C), indicating that both supplements induced a significant increase of the OXPHOS rate. This result did not surprise us since resveratrol, a polyphenol as catechins are, enhanced the ATP synthesis rate in OXPHOS-deficient primary cells that we recently assayed [41]. At variance, in both GTE and EGCG cardiomyocytes, the state 4 respiration rate was found to be similar to that in controls (Figure 1D). In addition, we estimated the respiratory control ratio: the state 3 to state 4 respiration rate ratio. This parameter strictly depends on both the functional integrity of the inner mitochondrial membrane and the OXPHOS complexes’ level/stoichiometry; therefore, it is considered an index of the OXPHOS efficiency. Indeed, the energetic coupling between the respiratory chain and the ATP synthase activities, estimated as RCR, was found to be almost unchanged in both GTE and EGCG cardiomyocytes compared to that in controls (Figure 1E).

The enzymatic activity of citrate synthase (CS) was then assayed to test whether the increase of ADP-dependent respiration rate (state 3) detected in both GTE and EGCG cardiomyocytes was due to mitochondrial mass increase. CS is a tricarboxylic acids cycle (TCA) enzyme located in the mitochondrial matrix only, and it is used as a mitochondrial mass index [42]. Indeed, no significant change of the CS activity was detected in both GTE and EGCG cardiomyocytes compared to that in controls (Figure 1F), thus suggesting that the increase of the state 3 respiration was likely due to an increase of the OXPHOS complexes’ level and/or activity.

### 3.2. GTE and EGCG Modulation of OXPHOS Complexes’ Level in Rat Cardiomyocytes

**Figure 2:**
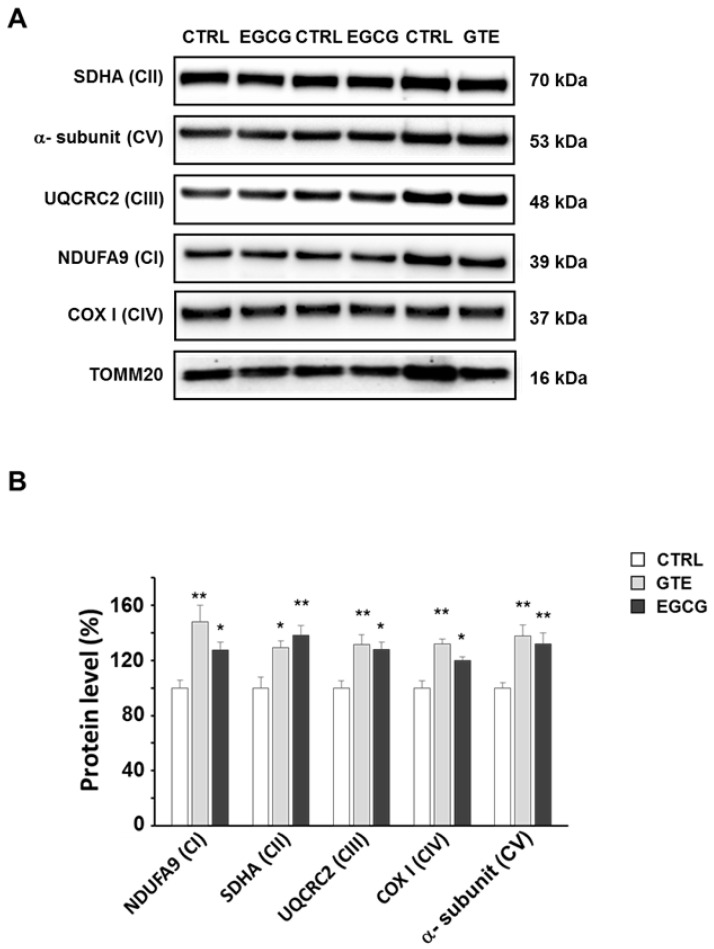
Oxidative phosphorylation (OXPHOS) complexes’ level in GTE and EGCG rat cardiomyocytes. (**A**) Typical electrophoretic separation and immunodetection of OXPHOS complexes in cell lysates of CTRL (*n* = 5), GTE- (*n* = 5), and EGCG-supplemented (*n* = 5) rats. (**B**) The subunit level of each OXPHOS complex was determined by means of the ImageLab analysis software and normalized to the translocase of outer mitochondrial membrane 20 (TOMM20) protein level, taken as an index of loading control. Histograms show the mean ± SEM; * *p* < 0.05 and ** *p*< 0.01 indicate the statistical significance of data compared to controls.

**Figure 3:**
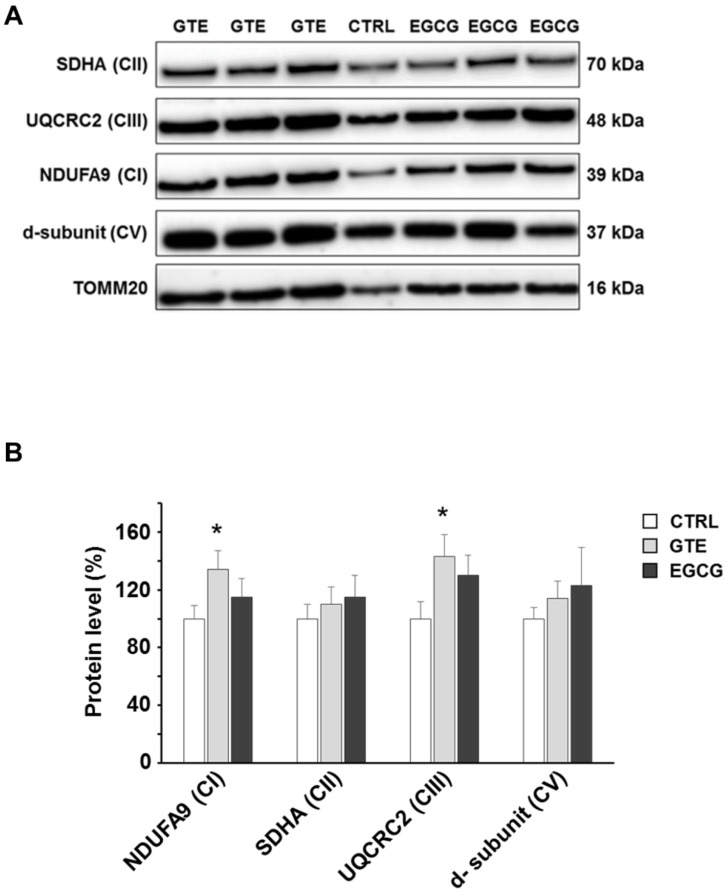
OXPHOS complexes level in GTE and EGCG rat LV tissue. (**A**) Typical electrophoretic separation and immunodetection of OXPHOS complexes in LV tissue lysates of CTRL (*n* = 2), GTE- (*n* = 3), and EGCG-supplemented rats (*n* = 3). (**B**) Subunit level of each OXPHOS complex was determined by means of the ImageLab analysis software and normalized to the TOMM20 protein level, taken as an index of loading control. Histograms show the mean ± SEM; * *p* < 0.05 indicates the statistical significance of data compared to controls.

To assess the levels of the OXPHOS complexes, cardiomyocytes were lysed and analyzed by SDS-PAGE. A specific subunit of each OXPHOS complex was identified by immunodetection and quantified by densitometric analysis (Figure 2A). To estimate the relative protein content, each subunit band was normalized to the mitochondrial protein loading control TOMM20. All the complexes of cardiomyocytes from both GTE- and EGCG-supplemented rats were found about 30% increased compared to those in controls, with the exception of complex I (CI) which was found to be nearly 50% higher in GTE rat cells (Figure 2B). In the LV heart tissue of both GTE and EGCG rats a significant enrichment of both CI (33% and 15%, respectively) and CIII (45% and 33%, respectively) was observed (Figure 3).

### 3.3. Effects of GTE and EGCG on mtDNA Content and Mitochondrial Biogenesis in Rat LV Heart Tissue

**Figure 4:**
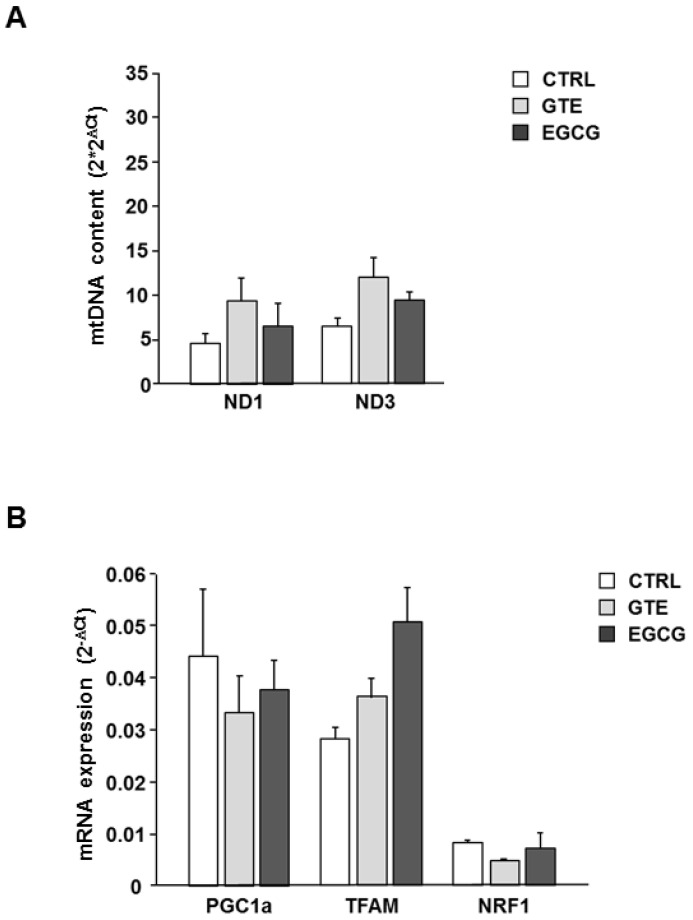
Effects of GTE and EGCG on LV heart tissue mtDNA content and mitochondrial biogenesis. (**A**) Relative mtDNA content measured in LV heart tissue of CTRL (*n* = 2), GTE- (*n* = 3), and EGCG-supplemented rats (*n* = 3). *ND1*, *ND3* region of the mtDNA were amplified. Data were normalized to the content of nuclear DNA, estimated by *GAPDH* amplification. On the Y axis 2 × 2^ΔCt^ ± SEM has been reported, where ΔCt = Ct _nucl gene_ − Ct _mito_ gene. (**B**) Relative expression of genes involved in mitochondrial biogenesis measured in LV heart tissue of CTRL (*n* = 2), GTE- (*n* = 3), and EGCG-supplemented rats (*n* = 3). On the Y axis 2^−^^ΔCt^ ± SEM has been reported, where ΔCt = Ct _target gene_ − Ct _GAPDH_.

The mtDNA content was measured in the LV tissue of hearts to measure the effect of both GTE and EGCG on mitochondrial mass. PCR amplification of mtDNA was performed in two distinct mitochondrial sequences that do not have duplicate pseudogenes in the nuclear genome. Specifically, amplicons of *ND1* and *ND3* were generated. Although the relative mtDNA content, calculated as mtDNA to nuclear DNA ratio, showed a slight trend to increase in both GTE and EGCG hearts compared to that in controls (Figure 4A), no statistically significant difference was observed among groups. Despite the small number of animals used for this experiment, the result of the analysis suggested that the improvement of mitochondrial function observed in the supplemented animals occurred without a significant increase of the mitochondrial mass. Consistently, the expression of genes involved in mtDNA replication and transcription did not change in the same tissue specimens (Figure 4B).

The absence of important changes of mitochondrial mass following GTE and EGCG treatment, as found by determining mtDNA content and transcription factors involved in mitochondrial biogenesis, was consistent with the observation carried out in isolated cardiomyocytes by means of an evaluation of the citrate synthase activity (Figure 1F).

**Figure 5:**
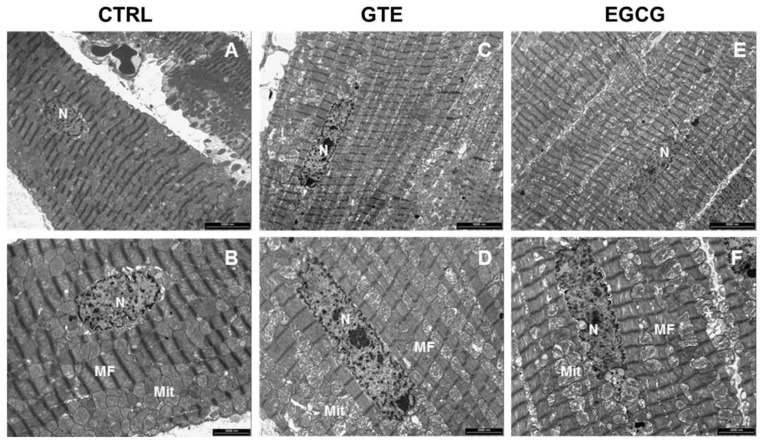
Transmission Electron Microscopy (TEM) analysis of the LV heart tissue. Representative images of the LV myocardium from CTRL (**A**,**B**) *n* = 1, GTE (**C**,**D**) *n* = 1, and EGCG (**E**,**F**) *n* = 1 rat hearts, illustrating at different magnification the distribution of mitochondria (Mit) and myofibrils (MF) in cardiomyocytes. *n*: nucleus. Scale bars = 5 µm (**A**,**C**,**E**); scale bars = 2 µm (**B**,**D**,**F**).

The ultrastructural features of the LV heart tissue from CTRL, GTE, and EGCG rat hearts are reported in Figure 5. In all of the groups, the cardiomyocytes exhibited well-aligned sarcomere striation (Figure 5A–F). However, interfibrillar and perinuclear mitochondrial engulfment was evident mostly in GTE- (Figure 5C,D) and, to a lesser extent, in EGCG-supplemented rats (Figure 5E,F).

### 3.4. Cardiomyocyte Mechanics and Calcium Transients

**Figure 6:**
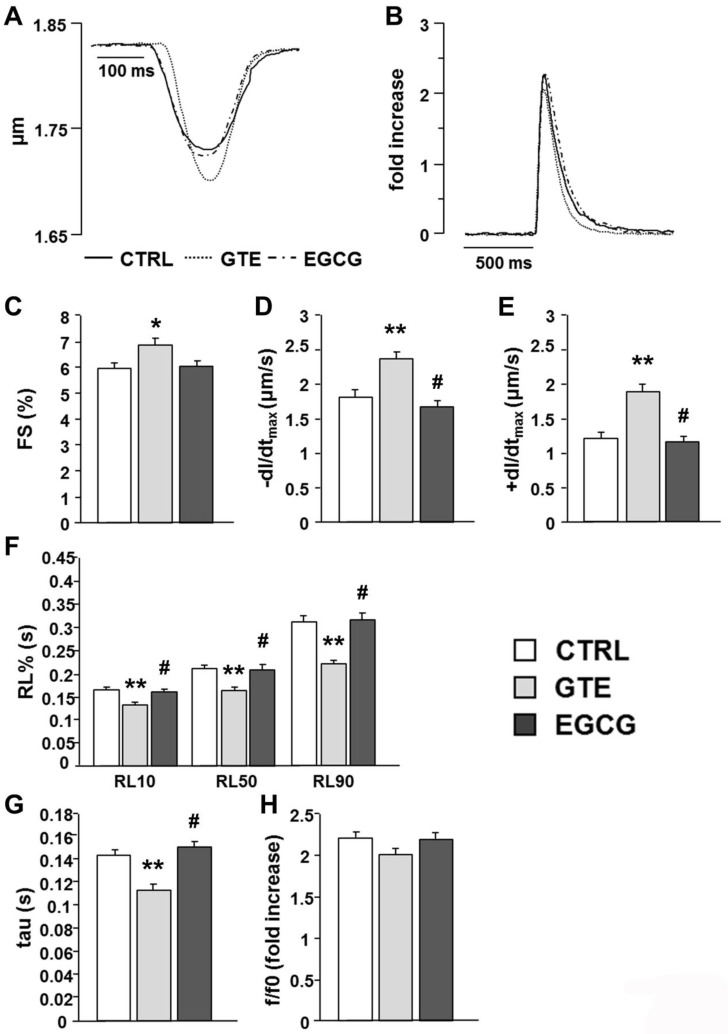
Effects of GTE or EGCG administration on cell mechanics and calcium transients. (**A**) Representative examples of sarcomere shortening and (**B**) calcium transients (traces were normalized and expressed as fold increase) recorded in ventricular myocytes from CTRL (*n* = 5), GTE (*n* = 5), and EGCG rats (*n* = 5). (**C**–**F**) Histograms show the mean ± SEM of the measurements performed in 66 CTRL, 72 GTE, and 78 EGCG cardiomyocytes: (**C**) fraction of shortening (FS); (**D**) maximal rate of shortening (−dl/dt_max_); (**E**) maximal rate of relengthening (+dl/dt_max_); (**F**) time to 10%, 50%, and 90% of relengthening (RL10%, RL50%, and RL90%); (**G**) fluorescence signal decay (tau); (**H**) amplitude of the calcium transient (f/f0). * *p* < 0.05, ** *p* < 0.01 indicate significant differences vs. CTRL; # *p* < 0.01 indicates significant differences between GTE and EGCG.

We then evaluated whether the increased mitochondrial performance induced by the two treatments had a functional counterpart in terms of cell mechanical properties. The average diastolic sarcomere length was similar in all groups, approximately 1.76 µm (average value: 1.755 ± 0.004 µm). Conversely, contractile efficiency and calcium dynamics measured in unloaded ventricular myocytes isolated from the GTE group were markedly enhanced in comparison with both CTRL and EGCG group (Figure 6A–H). GTE cardiomyocytes exhibited a significant increase in the fraction of shortening (FS, +15%; Figure 6C) and in the maximal rate of shortening (−dl/dt_max_, +31%; Figure 6D) and relengthening (+dl/dt_max_, +57%; Figure 6E), leading to a global reduction in relengthening times (RL10%, −24%; RL50%, −24%; RL90%, −29%; Figure 6F). Consistent with the cell motion data, the time required for cytosolic calcium removal significantly decreased (tau, −21%; Figure 6G). No difference was observed among the experimental groups in the amplitude of the Ca^2+^ transient (f/f0; Figure 6H).

### 3.5. Electrophoresis and Western Blot Analysis of SERCA2, PLB, and Phosphorylated Phospholamban (p-PLB)

**Figure 7:**
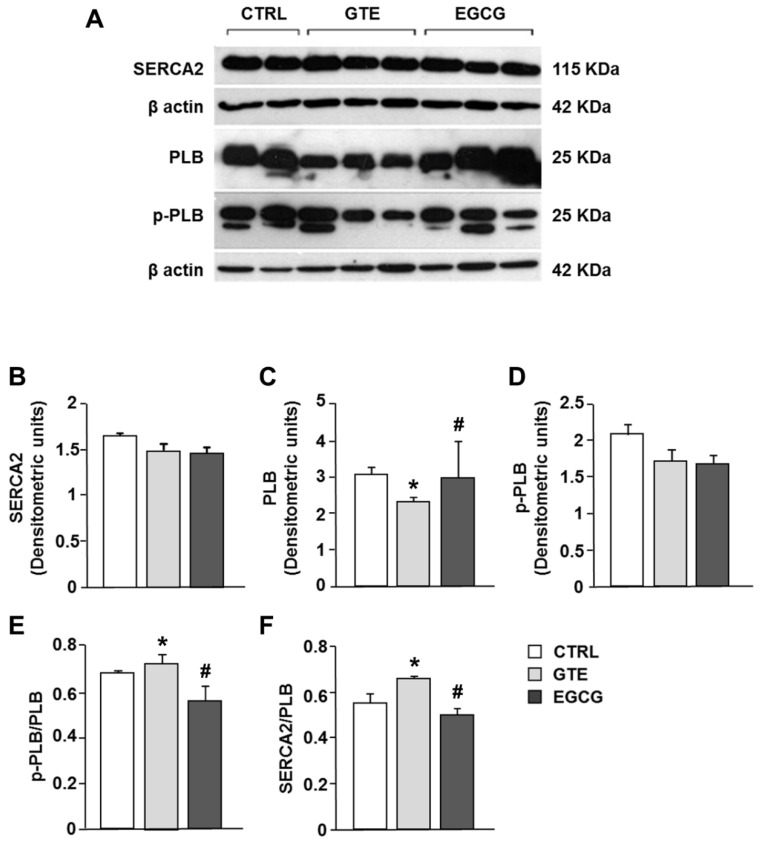
Sarcoplasmic reticulum calcium-ATPase (SERCA2), phospholamban (PLB), and phosphorylated phospholamban (p-PLB) protein expression changes in LV heart tissue of CTRL (*n* = 2), GTE- (*n* = 3), and EGCG-supplemented rats (*n* = 3). (**A**) Typical electrophoretic separation and immunodetection of SERCA2, PLB, p-PLB, in the LV heart tissue of control and supplemented rats. The expression levels of SERCA2 (**B**), PLB (**C**), and p-PLB (**D**) were measured by densitometric analysis and normalized to β-actin. Densitometry was performed by the Quantity One analysis software (Bio-Rad). p-PLB/PLB (**E**) and SERCA2/PLB (**F**) ratios. Histograms show the mean ± SEM. * *p* < 0.05 indicates significant differences vs. CTRL; # *p* < 0.05 indicates significant differences between GTE and EGCG.

The expression level of SERCA2 was comparable in the LV tissue of CTRL, GTE-, and EGCG-supplemented rats (Figure 7A,B). SERCA2 activity is mainly modulated by the small inhibitor protein phospholamban (PLB). We measured a significant decrease of PLB expression in GTE tissue samples as compared to that in both EGCG and CTRL tissue (Figure 7A–C), while p-PLB expression was comparable among all groups (Figure 7A–D). Accordingly, both SERCA2/PLB and p-PLB/PLB ratios were significantly increased in GTE tissue samples when compared to that in the other groups (Figure 7E,F; *p* < 0.05 vs. EGCG and CTRL).

## 4. Discussion

In the present study, we compared the effects of the 4-week oral administration of Theaphenon-E or EGCG, given at equivalent dosages, to healthy rats on both the morphology and functions of cardiomyocyte mitochondria and cell contractility.

Overall, our results showed that both GTE and EGCG promoted mitochondrial function, as documented by an increase of cell respiration rate under state 3 respiratory conditions and the consequent increase of cardiomyocyte energy availability (i.e., ATP). However, only the whole extract improved cell mechanical efficiency and intracellular calcium dynamics by affecting the expression of PLB, the protein that modulates the activity of SERCA2.

Although many studies investigated the molecular mechanisms underlying the beneficial effects of green tea catechins in vitro [2,7], to the best of our knowledge, the present study is the first to show that cardiomyocyte mitochondria are actually targets of both GTE and EGCG given at physiological doses, according to an oral administration schedule. This is particularly relevant, considering that catechins are absorbed to a limited extent through the gut and undergo an extensive metabolism that reduces their bioavailability. Both these issues are largely overlooked when the experiments are carried out in vitro using a supra-physiological amount of green tea catechins.

The mitochondria of the GTE and EGCG rat cardiomyocytes appeared mainly to be located at the interfibrillar and perinuclear regions, in the so-called condensed state [43,44], and were characterized by very dense matrix compartments (see Figure 5), suggesting a higher ADP phosphorylation competence compared to that in controls. Consequently, we hypothesized that this enhancement of the mitochondrial function could be due to enhanced levels of all the OXPHOS complexes (i.e., the four respiratory chain complexes and the ATP synthase) and/or to an increase of the mitochondrial mass. Indeed, by measuring the maximal respiration rate (state 3 respiration), we showed it was higher in both the GTE and the EGCG cardiomyocytes compared to that in controls. We also showed that the respiration rate increase was due to the enhanced level of all the OXPHOS complexes, suggesting that these cells could increase the oxidation of the heart energetic substrates, among which fatty acids are the most used. Therefore, our data match and support earlier findings that indicated the beneficial effects of tea catechins in stimulating lipid catabolism and fatty acid β-oxidation [45]. A significant increase of both complexes I and III was also observed in the LV tissue of supplemented rats as compared to that in controls. A possible reason for the absence of a significant increase of complexes II and IV in the LV tissue (at variance to isolated cardiomyocytes) can be easily explained by the cellular heterogeneity of the myocardium, consisting of fibroblasts and endothelial cells besides cardiomyocytes, which occupy only a fraction (about 70%) of the mammalian heart volume [46,47].

Regarding the cellular mitochondrial mass, we did not observe any significant effect of either supplement on it, as measured by the citrate synthase activity in isolated cardiomyocytes. This observation was also supported by the analysis of the mtDNA copy number, which is used as a surrogate biomarker of the mitochondria mass in cells [42,43,44,46,47,48], and by the expression of genes involved in mitochondrial biogenesis. Therefore, the improvement of cardiomyocyte bioenergetics that we observed does not seem to be explained by mitochondrial mass changes.

The cardiomyocyte contractile properties strongly depend on ATP availability [28,49]; therefore, the enhanced mitochondrial function induced by long-term administration of both GTE and EGCG was expected to positively affect them. However, we found that only GTE improves cardiomyocyte mechanics and intracellular calcium dynamics, suggesting that additional different intracellular pathways are promoted by the administration of the entire extract, rather than by administration of EGCG alone. In fact, in accordance with our previous observations [21], GTE cardiomyocytes exhibited an increased fraction of shortening associated with higher contraction–relaxation rates as compared to both control and EGCG-supplemented rats and, thus, an acceleration of cytosolic calcium removal. By contrast, EGCG given to animals at a dosage equivalent to that of GTE did not influence cell contractile/relengthening processes. The GTE-induced decrease of the phospholamban level resulted in a parallel increase of the SERCA2/PLB and p-PLB/PLB ratios as compared to those in both EGCG and CTRL, as is consistent with our previous results [21]. PLB, the small regulatory protein that modulates the active transport of Ca^2+^ by SERCA2 into the lumen of the sarcoplasmic reticulum, is a reversible inhibitor of SERCA2, and this inhibition is relieved upon phosphorylation, commonly due to beta-adrenergic stimulation and enhanced cAMP-dependent protein kinase A activity [50,51]. In this way, PLB modulates SERCA2-mediated myocardial relaxation during diastole. The reduction in PLB expression levels is known to be associated with a linear increase of the affinity of SERCA2 for Ca^2+^, the extent of myocyte cell shortening, and the rates of myocyte contraction and relengthening [51,52,53,54,55], as we actually observed. Furthermore, a hyperdynamic cardiac function was observed in PLB knockout mice, without any pathological or adverse functional consequences [52,53,54,55]. By contrast, PLB overexpression leads to decreased shortening fractions and rates of contraction and relaxation in murine cardiomyocytes [51].

In addition, the increase in the SERCA2/PLB ratio is reported to be critical for defining the affinity of SERCA2 for calcium [56,57], yielding positive effects on cardiomyocyte contractility, while a decreased ratio has the opposite effects. This is particularly evident at low frequencies [50,58,59]. Although we did not investigate the specific intracellular mechanisms leading to the downregulation of PLB expression in GTE cardiomyocytes, it should be considered that many dietary polyphenols, including catechins, can affect gene expression by epigenetic mechanisms through the modulation of different histone deacetylase activities [7,60,61,62]. The lack of effects on cellular mechanics of EGCG alone seems to suggest that the entire GTE is required to enhance the cardiomyocyte contractile efficiency. This is in line with previous studies showing that all the four main green tea catechins (EGC, EC, EGCG, and ECG) are capable of affecting the myocardial contractile efficiency to different extents [63,64,65,66,67].

## 5. Conclusions

In conclusion, we showed that a standardized GTE and an equivalent dose of EGCG as that contained in GTE, given in vivo to healthy rats, increased the mitochondrial function through the enhancement of the level of the OXPHOS complexes. In addition, the GTE only improved the cardiomyocyte mechanical performance by targeting excitation–contraction key proteins. We wonder whether GTE or EGCG might similarly affect tissues other than the myocardium. This cannot be excluded, since catechins have been found to act on many different tissues [68]. However, the heart is characterized by two main features considered in this study: a particularly high oxidative metabolism, fueled by fatty acid oxidation, and particularly intense Ca^2+^ dynamics. Therefore, it would be interesting to further investigate by assessing whether these observed effects may occur in those tissues characterized by an intense oxidative metabolism, such as the skeletal muscle. Concerning skeletal myocytes, where SERCA isoforms are highly expressed, it is reasonable to hypothesize that GTE might positively affect the cellular mechanical properties, through mechanisms similar to those observed in cardiomyocytes.

Our findings also suggest that GTEs, such as Theaphenon E, might be a valuable adjuvant tool for counteracting the occurrence and/or the progression of cardiomyopathies in which mitochondrial dysfunction, reduced energy availability, and changes of either expression or activity of the proteins responsible for the cardiomyocyte contractile efficiency [29,69,70,71,72] constitute early pathogenic factors. Further studies are required to verify this hypothesis on experimental models of cardiomyopathies in order to confirm the effectiveness of Theaphenon-E in reducing myocardial functional damage.

## Figures and Tables

**Table 1:** Body weights (g). Mean values ± standard error of the mean (SEM) of body weights measured once a week, in controls (CTRL) and green tea extract (GTE)- and epigallocatechin-3-gallate (EGCG)-supplemented animals.

	CTRL	GTE	EGCG
**Day 0**	423.2 ± 5.9	426.0 ± 16.4	427.2 ± 10.8
**Day 7**	428.4 ± 7.5	426.0 ± 16.9	425.2 ± 12.9
**Day 14**	431.4 ± 9.4	434.6 ± 19.2	436.0 ± 10.3
**Day 21**	430.8 ± 11.6	435.6 ± 15.9	441.8 ± 10.7
**Day 28**	437.0 ± 9.6	441.4 ± 21.2	441.2 ± 7.9

**Table 2:** Primer sequences.

Gene	Forward Primer Sequence 5′ - 3′	Reverse Primer Sequence 5′ - 3′
*GAPDH*	GTTCCAGAGACAGCCGCATC	CGTTCACACCGACCTTCACC
*PGC-1α* [37]	GTGCAGCCAAGACTCTGTATGG	GTCCAGGTCATTCACATCAAGTTC
*NRF* [38]	AAATTGGGCCACATTACAGGG	GTTGCATCTCCTGAGAAGCG
*TFAM* [38]	AGAGTTGTCATTGGGATTGG	CATTCAGTGGGCAGAAGTC
*ND1* [39]	TTAATTGCCATGGCCTTCCTCACC	TGGTTAGAGGGCGTATGGGTTCTT
*ND3* [38]	CAACAAGTTCTGCACGCCTTCCTT	TTGTTTGAATCGCTCATGGGAGGG
